# Diagnostic biomarker kinetics: how brain-derived biomarkers distribute through the human body, and how this affects their diagnostic significance: the case of S100B

**DOI:** 10.1186/s12987-022-00329-9

**Published:** 2022-05-11

**Authors:** Robert Murcko, Nicola Marchi, Damian Bailey, Damir Janigro

**Affiliations:** 1 FloTBI Inc., Cleveland, OH USA; 2 Department of Physiology and Biophysics, Case Western Reserve University, Cleveland, OH USA; 3 Laboratory of Cerebrovascular and Glia Research, Department of Neuroscience, Institute of Functional Genomics (UMR 5203 CNRS - U 1191 INSERM), University of Montpellier, Montpellier, France; 4 Neurovascular Research Laboratory, Faculty of Life Sciences and Education, University of South Wales, Newport, UK

**Keywords:** Computer model, MATLAB, Simbiology, Astrocytes, Physiologically-based pharmacokinetic model, Glymphatics, Extracranial sources, Traumatic brain injury, Brain barriers, Saliva

## Abstract

Blood biomarkers of neurological diseases are often employed to rule out or confirm the presence of significant intracranial or cerebrovascular pathology or for the differential diagnosis of conditions with similar presentations (e.g., hemorrhagic vs. embolic stroke). More widespread utilization of biomarkers related to brain health is hampered by our incomplete understanding of the kinetic properties, release patterns, and excretion of molecules derived from the brain. This is, in particular, true for S100B, an astrocyte-derived protein released across the blood–brain barrier (BBB). We developed an open-source pharmacokinetic computer model that allows investigations of biomarker’s movement across the body, the sources of biomarker’s release, and its elimination. This model was derived from a general in silico model of drug pharmacokinetics adapted for protein biomarkers. We improved the model’s predictive value by adding realistic blood flow values, organ levels of S100B, lymphatic and glymphatic circulation, and glomerular filtration for excretion in urine. Three key variables control biomarker levels in blood or saliva: blood–brain barrier permeability, the S100B partition into peripheral organs, and the cellular levels of S100B in astrocytes. A small contribution to steady-state levels of glymphatic drainage was also observed; this mechanism also contributed to the uptake of organs of circulating S100B. This open-source model can also mimic the kinetic behavior of other markers, such as GFAP or NF-L. Our results show that S100B, after uptake by various organs from the systemic circulation, can be released back into systemic fluids at levels that do not significantly affect the clinical significance of venous blood or salivary levels after an episode of BBB disruption.

**Supplementary Information:**

The online version contains supplementary material available at 10.1186/s12987-022-00329-9.

## Background

Pharmacokinetic in silico models are essential for pharmacological studies and drug development. During the drug discovery and development process, potential clinical candidates are screened for their absorption, distribution, metabolism, and excretion (ADME) properties to avoid clinic failures related to inappropriate ADME properties. Until recently, most pharmacokinetic models were aimed at predicting the properties of small (< 1 kD) molecules after oral or intravenous (i.v.) administration. Recently, biologics have become a significant portion of therapeutic agents, and the old small molecule software strategies had to be reformulated to adapt to large (> 10kD) molecular weight proteic therapeutics. While ad hoc software has been developed by Industry, academic efforts have used available platforms (e.g., MATLAB) to model how drugs distribute in the body.

A perhaps unexpected utilization of pharmacokinetic modeling of large, proteic agents is the development of modified strategies to study the movement of diagnostic molecules in the human body. Several of these biomarkers are proteins with varying molecular properties and sizes. These are, most commonly, not administered conventionally but are instead released or synthesized ex novo by a specific organ, neoplasm, or cell type. For example, troponins are proteins found in skeletal and cardiac muscle fibers that regulate muscular contraction. Troponin tests measure the level of cardiac-specific troponin in the blood to help detect heart injury [1]. When there is damage to heart muscle cells, troponin is released into the blood, thus becoming detectable by a simple blood test. The necessity of cellular death for biomarker release is not universal since many other biomarkers are released by healthy cells (see below for S100B). Our previous work has focused primarily on brain-derived diagnostic molecules used to diagnose CNS or neurological diseases. These include GFAP, S100B, UCHL-1, and other less-studied reporters of brain disease or health [2]. An example of how pharmacokinetic models can be applied to brain diagnostic markers was published [3].

We adapted and refined MATLAB-based models [3–5] for the present study using the published data obtained by real-life experiments (direct measurements of S100B from human tissues; see [3–5]). We specifically wished to explore the pharmacokinetics of S100B, a reporter of blood–brain barrier (BBB) dysfunction (BBBD) and brain health [6–9]. While several studies have promoted its use in neurology and psychiatry [3, 7, 9, 10], others expressed doubts about its reliability for human diagnostics. These concerns primarily derive from the pitfalls listed below.

It was suggested that S100B not only derives from the brain but also has extracranial sources [11–16]. Thus, when both brain and peripheral trauma are involved, it is impossible to dissect a central vs. peripheral origin of the biomarker. This is a pitfall in studies where S100B is elevated in individuals with multi-trauma of orthopedic nature [17]. The same issues were reported for other brain-derived biomarkers [18]. Several counterarguments have been made, showing, for example, that extracranial sources where S100B is synthesized from mRNA are few (e.g., testis, descending tubules in kidney [3]) and that S100B content in other organs derives from uptake from circulation [4]. It was recently shown that time-dependent internalization of circulating S100B by mesenchymal stem cells occurs via the pathways of clathrin- and lipid raft-mediated endocytosis [19]. Others have demonstrated that S100B in fat tissue does not contribute to peripherally detected levels [20], but the opposite was also suggested [21]. Therefore, controversy exists on the extent and relevance of extracranial sources of biomarkers used for CNS diagnostics.

In the field of sports medicine, it was shown that blood S100B increases after sub-concussive head hits [22, 23]: this was ascribed to increased BBB permeability as also documented by MRI [24]. Other studies have shown that S100B is increased by exercise alone [12, 25, 26], while others found no effect of strenuous exercise on S100B levels [27–30]. An explanation of these contrasting findings points to BBB damage induced by extreme exercise [2]. According to this hypothesis, strenuous exercise or performance in extreme sports results in BBB “opening,” possibly due to a mechanism involving free radical formation, as suggested by ref. [31]. In any case, it is not known how different sources of S100B contribute to the peripheral signal in blood (or saliva) [2, 26, 32, 33]. Lastly, a common motif in S100B diagnostics is that S100B is not specific for any neurological disease [3]. This is due to the fact that BBB leakage allowing S100B appearance in peripheral body fluids is a common feature of many neurological conditions [3].

Another point of contention relates to how the brain releases S100B during an insult. The leading hypothesis calls for a breach of the BBB as described above and in [34, 35]. An alternative hypothesis calls for the recently described glymphatic circulation as a means of brain release of biomarkers in blood [36]. The contribution of glymphatics in human subjects is unknown.

The scope of the present work is to answer, when possible, these questions by using two advanced full-body models of cerebrovascular and peripheral circulation after the release of S100B by the brain or other organs. A lymphatic compartment was also added to the model together with realistic urinary excretion pathways. The initial parameters of the model were derived from experimental observations and available human data [3].

## Methods

We used MATLAB 2019–2021b (MathWorks, Natick, MA) to design, test and simulate the model. The toolbox used was MATLAB’s Simbiology app (versions 5.0–6.2), aided by packages for partial differential equations, statistics, and parallel computing. Data were plotted in MATLAB and exported to CorelDraw (Corel Co.) as extended metafiles.

**Fig. 1:**
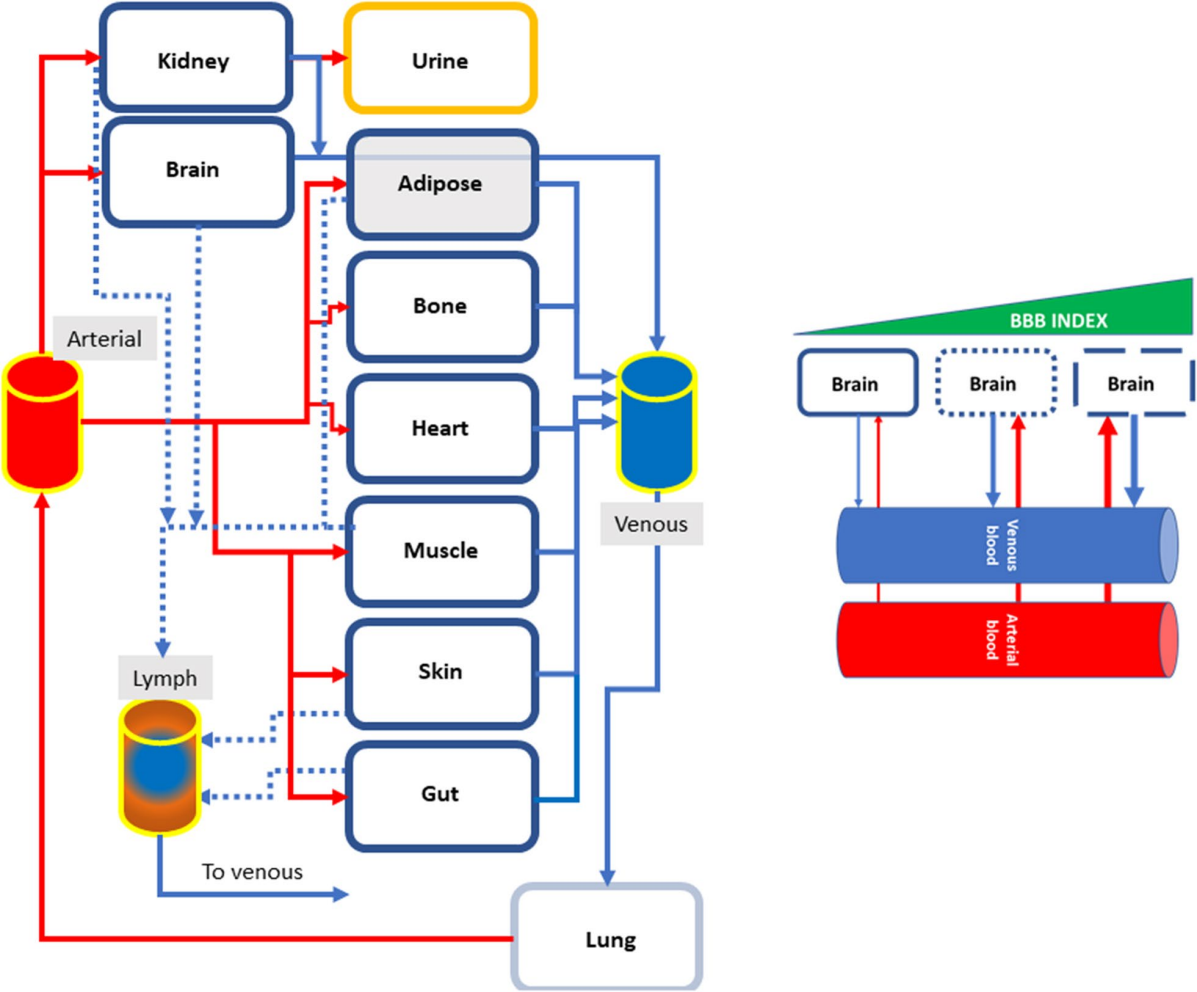
Schematic representation of the structure of Model 1. The *continuous red lines* depict flow through arteries (except for the lung), while the *continuous blue lines* refer to venous flow. The *dotted blue lines* show the lymphatic vessels connecting the organs directly to the venous compartment. The *right panel inset* shows a graphical representation of the mechanism of BBBD presented herein, underscoring that venous levels are greatly influenced by leakage of biomarkers from the brain into the circulation

We developed two separate and independent models to mimic the behavior of circulating brain-derived small molecular weight proteic biomarkers. Model 1 was used primarily to assess the relevance of various peripheral organs to the signal measured in blood (Fig. 1). Model 2 was developed after ruling out the contributions by heart, bone, and skin (Figs. 4 and 5): these organs are not included in Model 2, which uses a different set of equations to focus on the contributions of adipose, muscle, and gut tissues to measured levels of the biomarker in blood. Model 2 also adopts a more complex brain modeling, as detailed below. The following sections highlight the shared and specific modeling strategies used.

Most of the simulations shown were run to steady-state with or without an accompanying BBB disruption event. This allows to follow up the kinetics of S100B in each organ or compartments. For Fig. 10, the BBBD was triggered after steady-state was achieved.

### Model 1

A human full-body physiologically-based pharmacokinetic (PBPK) model was adapted from [37]. This model contains lung, brain, skin, bone, adipose tissue, heart, kidney, muscle, and gut (Fig. 1). The volume of these organs is specified in Table 1. The organs are connected by arterial and venous vessels, whose contributions to the vascular network are expressed in ml/hr (also listed in Table 1). The portal circulation was excluded for the sake of simplicity, nor were the spleen, thymus, and pancreas included. No data are available on their role related to the release and uptake of S100B or other markers of brain health. It was shown that the spleen contains S100B, but this expression was limited to CD4^+^/CD8^+^ immunocompetent cells [4].

**Table 1:** Parameter values used for Model 1 (Fig. 2)

Quantity name	Initial value	Units
Arterial blood	2500.00	Milliliter
S100B	0.00	Nanogram/milliliter
Brain	1340.00	Milliliter
S100B	9.00	Nanogram/milliliter
Kidney	325.00	Milliliter
S100B	0.25	Nanogram/milliliter
Urine	600.00	Milliliter
S100B	0.00	Nanogram/milliliter
Venous blood	2500.00	Milliliter
S100B	0.00	Nanogram/milliliter
Lung	547.00	Milliliter
S100B	0.37	Nanogram/milliliter
Heart	359.00	Milliliter
S100B	0.28	Nanogram/milliliter
Bone	3950.00	Milliliter
S100B	0.00	Nanogram/milliliter
Adipose tissue	22,700.00	Milliliter
S100B	0.51	Nanogram/milliliter
Skin	3150.00	Milliliter
S100B	0.28	Nanogram/milliliter
Muscle	31,300.00	Milliliter
S100B	0.30	Nanogram/milliliter
Gut	1220.00	Milliliter
S100B	0.40	Nanogram/milliliter
Lymph	12.00	Liter
S100B	0.00	Nanogram/milliliter
Muscle tissue vascular fraction	845.10	Milliliter
S100B	0.00	Nanogram/milliliter
Gut tissue vascular fraction	61.00	Milliliter
S100B	0.00	Nanogram/milliliter
Lung tissue vascular fraction	101.20	Milliliter
S100B	0.00	Nanogram/milliliter
Heart tissue vascular fraction	15.08	Milliliter
S100B	0.28	Nanogram/milliliter
Bone tissue vascular fraction	197.50	Milliliter
S100B	0.00	Nanogram/milliliter
Kidney tissue vascular fraction	22.75	Milliliter
S100B	0.00	Nanogram/milliliter
Skin tissue vascular fraction	157.50	Milliliter
S100B	0.00	Nanogram/milliliter
Adipose tissue vascular fraction	703.70	Milliliter
S100B	0.00	Nanogram/milliliter
Brain tissue vascular fraction	67.00	Milliliter
S100B	0.00	Nanogram/milliliter
BloodFlowLungToArtery	313,980.00	milliliter/hour
BloodFlowVenousToLung	313,980.00	Milliliter/hour
BloodFlowArteryToKidney	66,000.00	Milliliter/hour
BBB_Index	0.00	Dimensionless
GFR	125.00	Milliliter/minute
BloodFlowArteryToSaliva	0.00	1/hour
BloodFlowArteryToVenous	3139.00	Milliliter/hour
BloodFlowArteryToMuscle	45,000.00	Milliliter/hour
BloodFlowArteryToHeart	9000.00	Milliliter/hour
BloodFlowHeartToVenous	9000.00	Milliliter/hour
BloodFlowArterialToBone	15,000.00	Milliliter/hour
BloodFlowBoneToVenous	15,000.00	Milliliter/hour
BloodFlowKidneyToVenous	66,000.00	Milliliter/hour
BloodFlowAdiposeToVenous	15,600.00	Milliliter/hour
BloodFlowArterialToAdipose	15,600.00	Milliliter/hour
BloodFlowSkinToVenous	18,000.00	Milliliter/hour
BloodFlowArterialToSkin	18,000.00	Milliliter/hour
TissueFactor	0.18	Dimensionless
BloodFlowMuscleToVenous	45,000.00	Milliliter/hour
BloodFlowArteryToGut	66,000.00	Milliliter/hour
BloodFlowGutToVenous	66,000.00	Milliliter/hour
LymphFlowMuscle	90.00	Milliliter/hour
LymphFlowGut	132.00	Milliliter/hour
LymphFlowHeart	132.00	Milliliter/hour
LymphFlowAdipose	31.20	Milliliter/hour
LymphFlowSkin	36.00	Milliliter/hour
LymphFlowKidney	132.00	Milliliter/hour
LymphFlowToVein	3100.00	Milliliter/hour
kf_1	1.00	Dimensionless
BloodFlowLungToArterial	313,980.00	Milliliter/hour
Excretion	0.00	1/minute
RenalEliminationFactor	100.00	Dimensionless
kf_brain	0.02	Dimensionless
AdiposeTissueFactor	1.00	Dimensionless
PoreRatioMuscle	2000.00	Dimensionless
PoreRatioAdipose	500.00	Dimensionless
PoreRatioGut	500.00	Dimensionless
PoreRatioSkin	500.00	Dimensionless
PoreRatioLung	45.00	Dimensionless
PoreRatioKidney	200.00	Dimensionless
kr_brain	0.00	Dimensionless
kf_muscle	24.44	Dimensionless
kr_muscle	0.00	Dimensionless
kf_gut	104.17	Dimensionless
kr_gut	0.01	Dimensionless
kf_lung	617.28	Dimensionless
kr_lung	0.06	Dimensionless
kf_heart	130.21	Dimensionless
kr_heart	0.01	Dimensionless
kf_bone	797.10	Dimensionless
kr_bone	0.08	Dimensionless
kf_kidney	135.14	Dimensionless
kr_kidney	0.01	Dimensionless
kf_skin	66.67	Dimensionless
kr_skin	0.01	Dimensionless
kf_adipose	57.14	Dimensionless
kr_adipose	0.01	Dimensionless
interstitialFlow	1.00	Milliliter/hour
BloodFlowFromBrain	42,000.00	Milliliter/hour
BloodFlowToBrain	42,000.00	Milliliter/hour

**Fig. 2:**
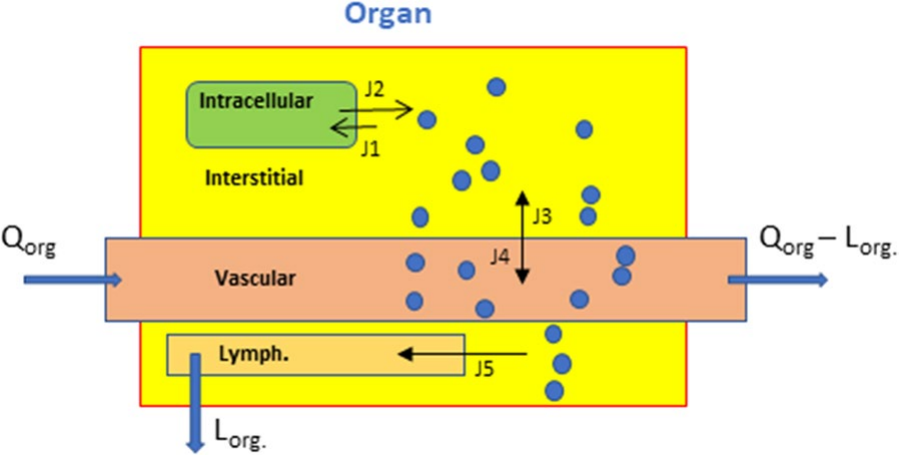
Structure of a single organ in Model 1. Note that two equilibria reactions (K_r_ and K_f_) describe the passage of biomarkers from the organ’s parenchyma (interstitial space) to blood and vice versa. A lymphatic vessel is also depicted. *Qorg* and *Lorg* refer to the blood flow into and out of the organ, and lymphatic flow, respectively. The values of Q for each organ are listed in Table 1

The initial levels of S100B (ng/ml) in each organ were derived from our previous work based on actual measurements [3, 4]. Each organ in the model contains a vascular fraction, i.e., an interface between parenchyma and vascular space (Fig. 2). The volume of the vascular fraction was obtained from [37]. The circulatory arterial-venous loop did not involve the heart and pulmonary circulation but rather consisted of a path through the lung (Fig. 1). A lymphatic circulatory system was added to all tissues; we modeled a central lymph collection where each lymphatic vessel out of tissue collects before drainage into venous blood.

The primary source of S100B in the body is the brain [38]. In our model, brain release of S100B into circulation is controlled by the variable *BBB_Index*. This dimensionless value varies from 0 to 1 (except in Fig. 10), reflecting no permeability across an intact BBB or "BBB opening," respectively.

1 $$\frac{{d\left( {Brain.S100B} \right)}}{dt} = \frac{1}{Brain}*\left( {\left( \begin{gathered} kf_{-\!\!-} brain{*}Brain.S100B - kr_{-\!\!-} brain\, \hfill \\ {*}\,\left\{ {Brain\,tissue\,vascular\,fraction} \right\}.S100B \hfill \\ \end{gathered} \right){*}\,interstitial\,Flow\,{*}\,BBB_{-\!\!-} Index} \right)$$

where *kf_brain* and *kr_brain* are dimensionless constants obtained based on the two-pore model as per references [37, 39]. Due to their size and polarity, protein biomarkers have limited direct diffusion across endothelial cell membranes. The fluid and protein movement occurs mainly by diffusion and convection through pores in the endothelial wall, which is limited by protein size. Data sources were gathered from [37] to determine kr and kf values for model 1. Small pore radii and large pore radii values for various tissue types were noted. Additionally, the ratio of small pore count to large pore count for that tissue type was noted, also provided in [37].

Using the data gathered, a ratio was taken to determine the magnitude of differences between the total amount of large pore radii within a tissue versus the total amount of small pore radii within a tissue. The equation used was:

1A $$kr_{-\!\!-} organ{ } = \frac{Large\,Pore\,Radius\,Size}{{Small\,Pore\,Radius\,Size\,{*}\,Ratio\,of\,Small\,Pores\,to\,Large\,Pores}}$$

To create a more pronounced differential within each tissue but keep the ratio of k values between each tissue standardized, the kf value was the kr value multiplied by a factor of 10,000, thus kf_organ = kr_organ * 10,000. This value was empirically chosen to match the rank order results for levels of S100B measured in various organs [3]. The order of measured values was brain > adipose > kidney > heart > muscle > lung > gut. The multiplier was derived by running the model with appropriate values to match the rank order of measured values. These values were then used as initial conditions.

The general equation for the organ’s uptake or release of protein biomarkers was:

2 $$\frac{{d\left( {Organ.S100B} \right)}}{dt} = \frac{1}{Organ}*\left( { - \left( {\left( \begin{gathered} kf_{-\!\!-} Organ\,{*}\,Organ.S100B - kr_{-\!\!-} Organ \hfill \\ *\left\{ {Organ\,Tissue\,Vascular\,Fraction} \right\}.S100B \hfill \\ \end{gathered} \right)*interstitial\,Flow} \right)} \right)$$

where the value *Interstitial flow* represents the flow rate of the protein within the organ, *Organ* and *Tissue Vascular Fraction* volume*s* were derived from ref. [37]. Organ.S100B refers to the concentration of S100B within the specified organ. In previous and subsequent equations all of these variables (Organ, Organ.S100B, Organ Tissue Vascular Fraction, etc.) are labeled with tags to the specific organ that they are referring to.

Excretion of the biomarker protein was modeled by kidney filtration:

3 $$\frac{{d\left( {Organ.S100B} \right)}}{dt} = \frac{1}{Urine}*\left( {\left( {Renal\,Elimination\,Factor\,{*}\,\frac{GFR}{{Kidney}}*Kidney.S100B} \right)*Kidney} \right)$$

where *GFR* is the glomerular filtration rate, and the *Renal elimination factor* is an additional dimensionless tuning parameter ranging from 0 to 1.

**Fig. 3:**
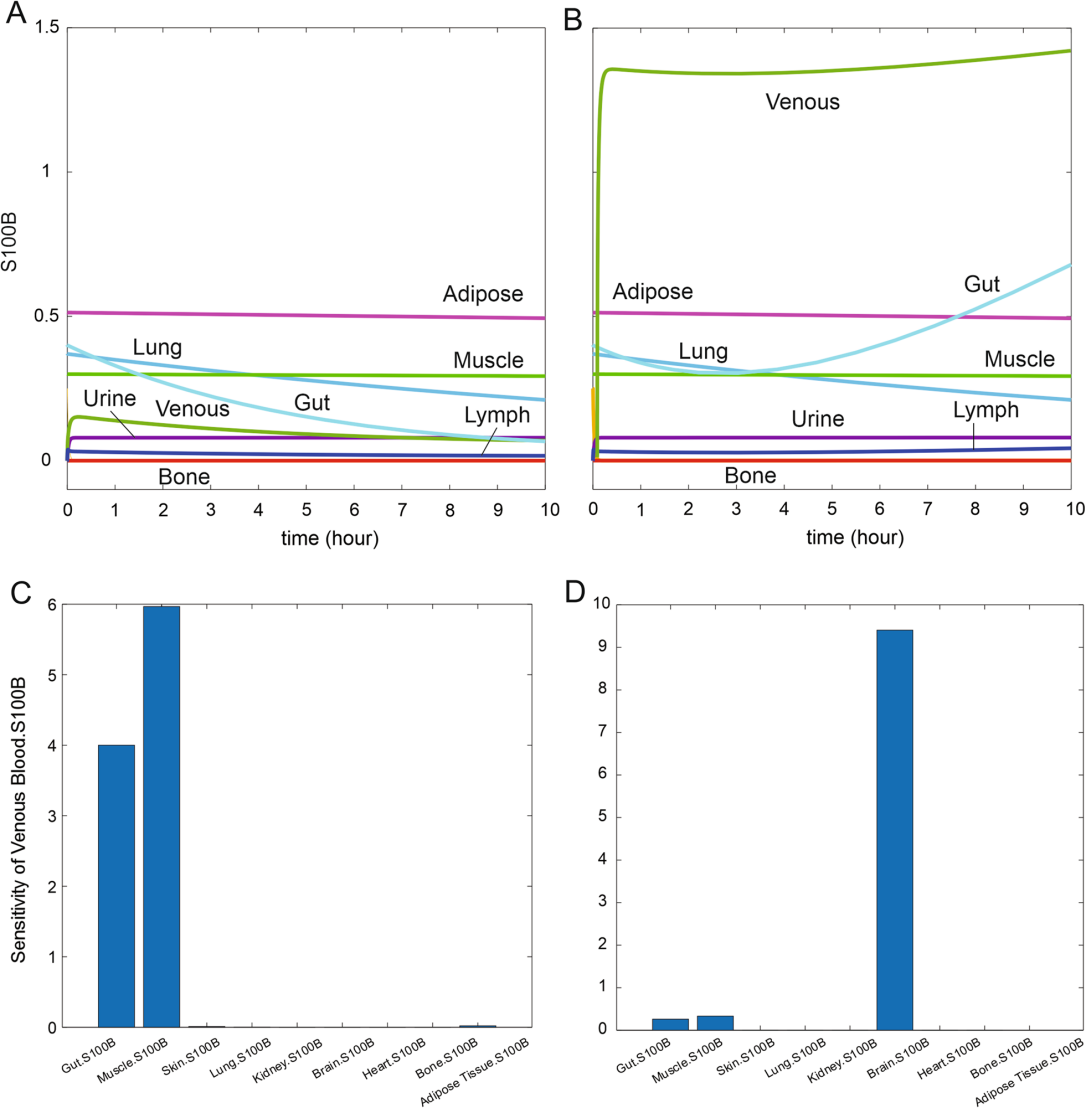
Results from simulations and sensitivity analysis (Model 1). The *left panel* shows the actual levels of S100B in organs before (**A** BBBD = 0) and after BBB disruption (**B**) obtained by setting the *BBB_Index* value to 1. Note the pronounced increase of S100B in venous blood with comparably smaller changes in other compartments. Figures **C** and **D** show the results of sensitivity analysis queries under the same conditions. Note that before BBBD (**A**) venous levels at steady state never reached the 0.1 ng/ml thresholds, the upper ceiling for control values of S100B. The x axis in the bar plots specifies the sensitivity inputs and y axes the sensitivity outputs integrated over time

Sensitivity analysis is the study of how the uncertainty in the output of a mathematical model can be divided and allocated to different sources of uncertainty in its inputs. In Simbiology, the routine of sensitivity analysis allows determining which rate constants and concentrations in a model significantly influence the overall behavior of the model (https://www.mathworks.com/help/simbio/ug/global-local-sensitivity-analysis-gsa-lsa-simbiology.html). SimBiology supports two types of sensitivity analyses: local and global sensitivity analysis (GSA). GSA uses Monte Carlo simulations, where a representative (*global*) set of parameter sample values are used to explore the effects of variations in model parameters of interest on the model response. In this approach, SimBiology performs a decomposition of the model output (response) variance by calculating the first- and total-order Sobol indices. The first-order Sobol indices give the fractions of the overall response variance that can be attributed to variations in an input parameter alone. The total-order Sobol index gives the fraction of the overall response variance that can be attributed to joint parameter variations (see [40]). We used global sensitivity analysis to interpret the impact of S100B in various organs on venous biomarker levels (Figs. 3 and 6; See also Additional file 1: Fig. S1, Additional file 2: Fig. S2, Additional file 3: Fig. S3). In Model 1, sensitivity analyses were run with *BBB_Index* set to either 0 or 1 (Fig. 3C and D, respectively). For Model 2, we explored the effect of changing S100B in the brain interstitium (1 and 10 ng/ml). The data in Supplemental figures were obtained by a Sobol sampling interpolation method, with 1000 samples; the simulation was run to steady state. The data are shown as time course (Additional file 1: Fig. S1, Additional file 2: Fig. S2) or bar graphs (Additional file 3: Fig. S3).

### Model 2

**Fig. 4:**
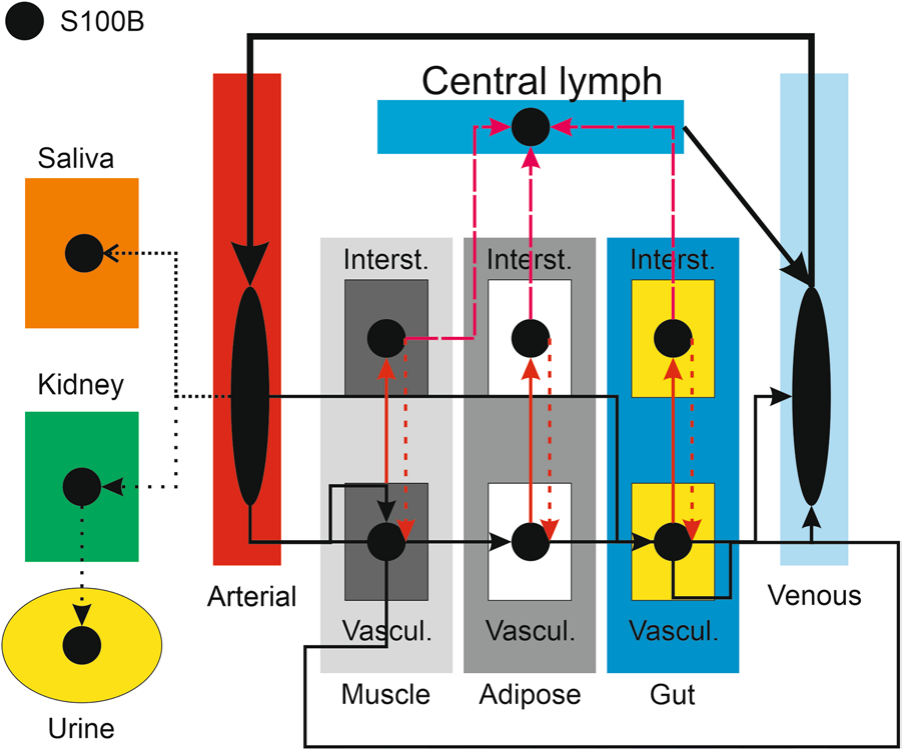
Schematic representation of Model 2. Note the addition of a salivary and glymphatic component. Peripheral organs are subdivided into interstitial and vascular compartments, while the brain is represented by three compartments, see Fig. 5. At the beginning of our simulations, S100B values in venous and lymphatic compartments were set to 0

**Fig. 5:**
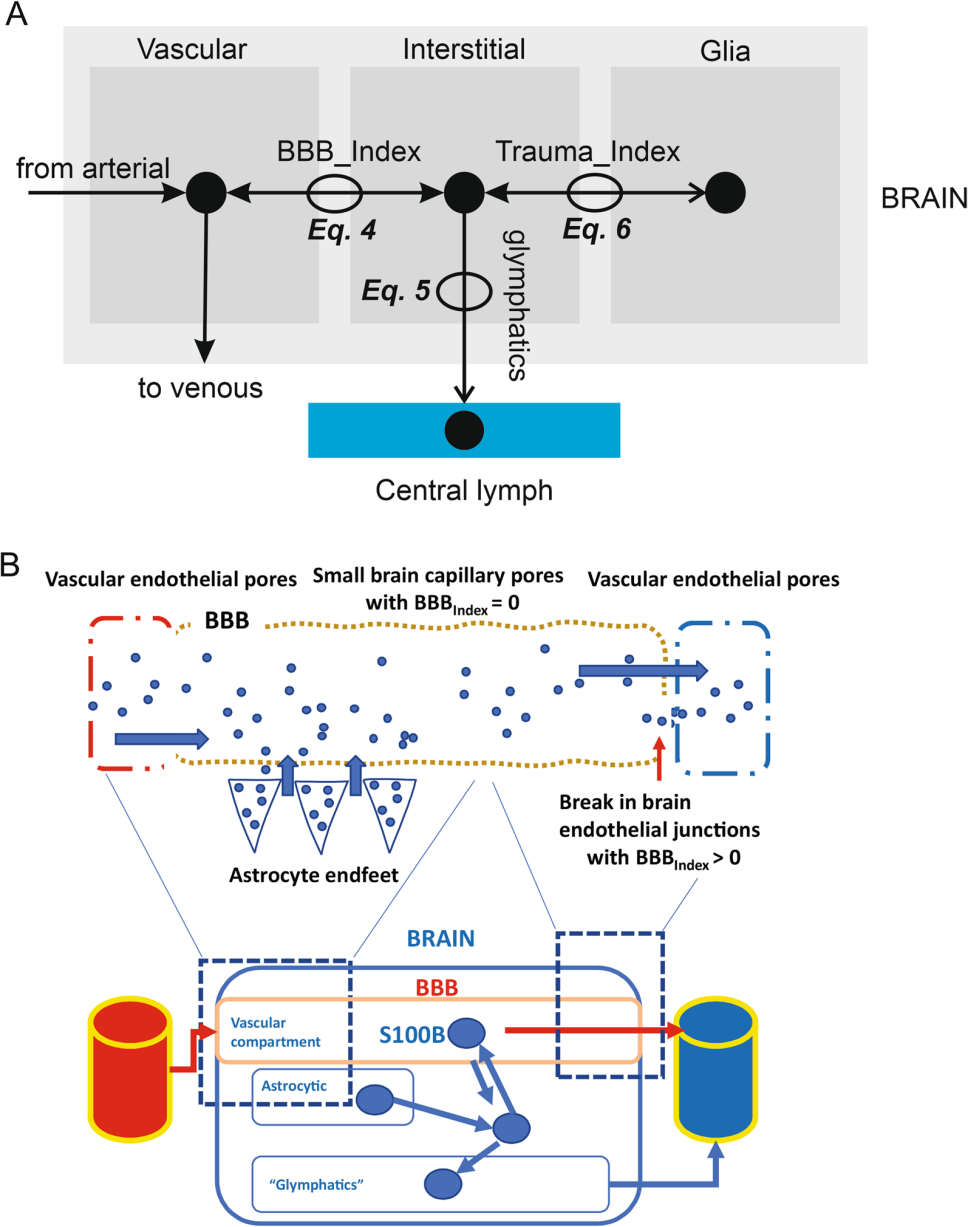
**A** Modeling of the CNS and its communications with the periphery. Note the equation numbers referring to the Methods. **B** Representation of the physiological reality to be modeled and schematics of the CNS components of the model. The *dotted lines* refer to the permeability of the BBB which is controlled by the *BBB_Index.* The Glia compartment is at equilibrium with the interstitial levels of S100B via the *Trauma_Index*

Model 2 follows the general structural backbone of Model 1 (Fig. 4). However, organs (except for the kidney, see below) are subdivided into vascular and interstitial compartments (Table 2). To describe the passage of protein from the interstitial (parenchymal) space into vascular space, we used the coefficient of vascular reflection (Sigma, or σ) as per reference [41]. The size-dependent restriction of large pores and small pores can, in fact, be represented as the vascular reflection coefficient, an indirect measure of the density of exchange pores. The model used to mimic the brain (Fig. 5) used *BBB_Index* and *Trauma_Index* to describe the passage of S100B across the interstitial, vascular, and cellular compartments. Note that unlike the dimensionless rate constants in Model 1, kinetic variables have dimensions of quantity/time in this model. The equation governing changes of biomarker’s levels in the vascular compartment was:

4 $$\begin{aligned} \frac{{d\left\{ {Vascular\,Compartment\,Brain} \right\}.S100B}}{dt} =\,& \frac{1}{Vascular\,Compartment\,Brain} \\ & *\,\left( {\left( {Arterial\,To\,Brain\,Blood\,Flow} \right)*\left\{ {Arterial \,Blood} \right\}.S100B} \right) \\ & + \left( {\left( {BBB_{-\!\!-} Index*\left\{ {Interstitium\,Brain} \right\}.S100B} \right)*\left\{ {Interstitium\,Brain} \right\}} \right) \\ \end{aligned}$$

where *BBB_Index* can change between 0 and 1 to mimic increased permeability of the cerebral vasculature. In addition to having a three compartment structure, brain modeling also included glymphatic drainage into central lymph and venous blood. The equation for brain interstitium S100B was thus:

5 $$\frac{{d\left( {\left\{ {Interstitium{ }Brain} \right\}.S100B} \right)}}{dt}{ } = \frac{{{ }1}}{{\left\{ {Interstitium\,Brain} \right\}}}{*}\left( \begin{gathered} \left( {\left( {Trauma_{-\!\!-} Index{*}Glia.S100B} \right){*}Glia} \right) \hfill \\ - \left( {Glymphatics{*}\left\{ {Interstitium\,Brain} \right\}.S100B} \right) \hfill \\ - \left( {\left( {BBB_{-\!\!-} Index{*}\left\{ {Interstitium\,Brain} \right\}.S100B} \right){*}\left\{ {Interstitium\,Brain} \right\}} \right) \hfill \\ \end{gathered} \right)$$

where the term *Trauma_Index* refers to the passage of S100B from astrocytes in the cellular compartment (Glia) released directly into the brain interstitium. *Glymphaticds* is the rate of interstitial flow to *Central lymph*. Changes of S100B in the cellular compartment were described by:

6 $$\left\{ {Interstitium\,Brain} \right\}.S100B = Glia.S100B*Trauma_{-\!\!-} Index$$

**Table 2:** Parameter values used for Model 2 (Fig. 4)

Quantity name	Initial value	Units
Arterial blood	1.16	Liter
S100B	0.00	Nanogram/milliliter
Venous blood	2.33	Liter
S100B	0.00	Nanogram/milliliter
Central lymph	12.00	Liter
S100B	0.00	Nanogram/milliliter
Brain	1.34	Liter
Vascular compartment brain	67.00	Milliliter
S100B	0.00	Nanogram/milliliter
Interstitium brain	130.00	Milliliter
S100B	10.00	Nanogram/milliliter
Glia	0.20	Liter
S100B	50.00	Nanogram/milliliter
Urine	200.00	Milliliter
S100B	0.00	Nanogram/milliliter
Kidney	280.00	Milliliter
S100B	0.25	Nanogram/milliliter
Muscle	31.30	Liter
Interstitium muscle	2.80	Liter
S100B	0.30	Nanogram/milliliter
Vascular space muscle	0.85	Liter
S100B	0.00	Nanogram/milliliter
Adipose	22.70	Liter
Interstitium adipose	3.20	Liter
S100B	0.50	Nanogram/milliliter
Vascular space adipose	703.10	Milliliter
S100B	0.00	Nanogram/milliliter
Gut	1220.00	Milliliter
Vascular space gut	61.00	Milliliter
S100B	0.00	Nanogram/milliliter
Interstitium gut	325.00	Milliliter
S100B	0.18	Nanogram/milliliter
Saliva	10.00	Milliliter
S100B	0.00	Nanogram/milliliter
ArterialToBrainBloodFlow	42,000.00	Milliliter/hour
BrainToVenousBloodFlow	42,000.00	Milliliter/hour
BBB_index	0.00	1/hour
Trauma_index	0.00	1/hour
Glymphatics	8.40	Milliliter/hour
VenousToArterialBloodFlow	201,874.29	Milliliter/hour
LymphaticToVenous	100.00	Milliliter/hour
GFR	10.00	Milliliter/hour
ArterialToKidneyBloodFlow	66,000.00	Milliliter/hour
EliminationFactor	100.00	1/minute
KidneyToVenousBloodFlow	66,000.00	Milliliter/hour
ReverseSigmaGut	0.05	1/hour
ReverseSigmaAdipose	0.05	1/hour
ReverseSigmaMuscle	0.05	1/hour
BBB	100.00	1/minute
SalivaryBloodFlow	1.00	Milliliter/minute
EmptyingSaliva	1.00	Dimensionless
kf	1.00	Dimensionless
kf_1	1.00	Dimensionless
SigmaBrainLymphatic	0.10	Dimensionless
TissuePartitionKidney	0.10	1/minute
ArterialToMuscleBloodFlow	45,000.00	Milliliter/hour
MuscleToVenousBloodFlow	45,000.00	Milliliter/hour
SigmaMuscleInterstitiumVascular	0.05	1/hour
MuscleLymphFlow	90.00	Milliliter/hour
SigmaMuscleLymphatic	0.80	Dimensionless
SigmaAdiposeInterstitiumVascular	0.05	1/hour
ArterialToAdiposeBloodFlow	15,600.00	Milliliter/hour
AdiposeToVenousBloodFlow	15,600.00	Milliliter/hour
SigmaAdiposeLymphatic	0.80	Dimensionless
AdiposeLymphFlow	31.20	Milliliter/hour
GutToVenousBloodFlow	66,000.00	Milliliter/hour
ArterialToGutBloodFlow	66,000.00	Milliliter/hour
SigmaGutInterstitiumVascular	0.05	1/hour
SigmaGutLymphatic	0.80	Dimensionless
GutLymphFlow	132.00	Milliliter/hour

*Glia.S100B* was set constant at 50 ng/ml as per in vitro measurements by others [42].

The kidney was modeled by a single compartment with input from arterial blood and an output to urine. The process was described by:

7 $$\frac{{d\left( {Kidney.S100B} \right)}}{dt} = \frac{ 1}{{Kidney}}*\left( \begin{gathered} - \left( {\left( {Elimination\,Factor*\frac{GFR}{{Kidney}}*\frac{Kidney.S100B}{{Tissue\,Partition\,Kidney}}} \right)*Kidney} \right) \hfill \\ + \left( {Arterial\,To\,Kidney\,Blood\,Flow*\left\{ {Arterial\,Blood} \right\}.S100B} \right) \hfill \\ - \left( {Kidney\,To\,Venous\,Blood\,Flow*Kidney.S100B} \right) \hfill \\ \end{gathered} \right)$$

and by:

8 $$\frac{{d\left( {Urine.S100B} \right)}}{dt} = \frac{ 1}{{Urine}}*\left( {\left( {\left( {Elimination\,Factor*\frac{GFR}{{Kidney}}*\frac{Kidney.S100B}{{Tissue\,Partition\,Kidney}}} \right)*Kidney} \right) - \left( {kf*Urine.S100B} \right)} \right)$$

*Tissue partition kidney* was set at 0.1/min. GFR was set at 10 ml/h; this non-constant value was explored during simulations (e.g., Fig. 8).

Note that in all figures, except Fig. 10, the simulation started before steady-state conditions were reached, thus allowing the variables to express the kinetic significance of the underlying code. See, for example, Fig. 3A, B, where the time-dependent changes in S100B are shown.

## Results

The structure of Model 1 is shown in Fig. 1, together with the graphic rendition of the process of BBB disruption. Figure 2 shows the formalism used to describe each organ in Model 1. The simulation of Model 1 led to the results shown in Fig. 3, which represents the changes in organs’ S100B levels under normal conditions (A, BBBD = 0) or after BBB disruption (B; *BBB_Index* = 1). Without BBB disruption, individual organs displayed a change in parenchymal S100B content to eventually reach steady state. Also, note that venous levels, at steady state, were < 0.1 ng/ml, which is consistent with clinical studies in normal adults when using the Roche Diagnostics test [43, 44]. Panels C and D show the results of the simulation in A and B processed for sensitivity analysis (see “Methods” section). The main contributors to venous blood levels were muscle and gut tissues, with minor contributions by adipose, lung, and skin. After BBB disruption, sensitivity analysis pointed to brain sources as primary contributors to venous levels.

Since only a few organs contributed to the overall venous signal, we developed Model 2 based on three organs (muscle, adipose, and gut) plus the kidney and a “virtual” urine container mimicking the bladder (Fig. 4). Salivary production was also added to the model. The main difference between the two models is the description of brain S100B movements within and outside the brain parenchyma. For the brain, three compartments were used: *vascular* (i.e., the cerebrovascular circulation), *interstitial* (the brain extracellular space), and *glia*, referring to astrocytes, the primary cell type expressing S100B in the body (Fig. 5). The correspondence of the model with brain physiology is shown in Fig. 5B. In addition to the arterial influx and venous efflux, a glymphatic distribution process draining into Central lymph was added to the model. Another difference in Model 2 is that the structure of the organs and S100B movements within was based on the reflection coefficient (Sigma) rather than two-pore theory calculations (see “Methods” section).

**Fig. 6:**
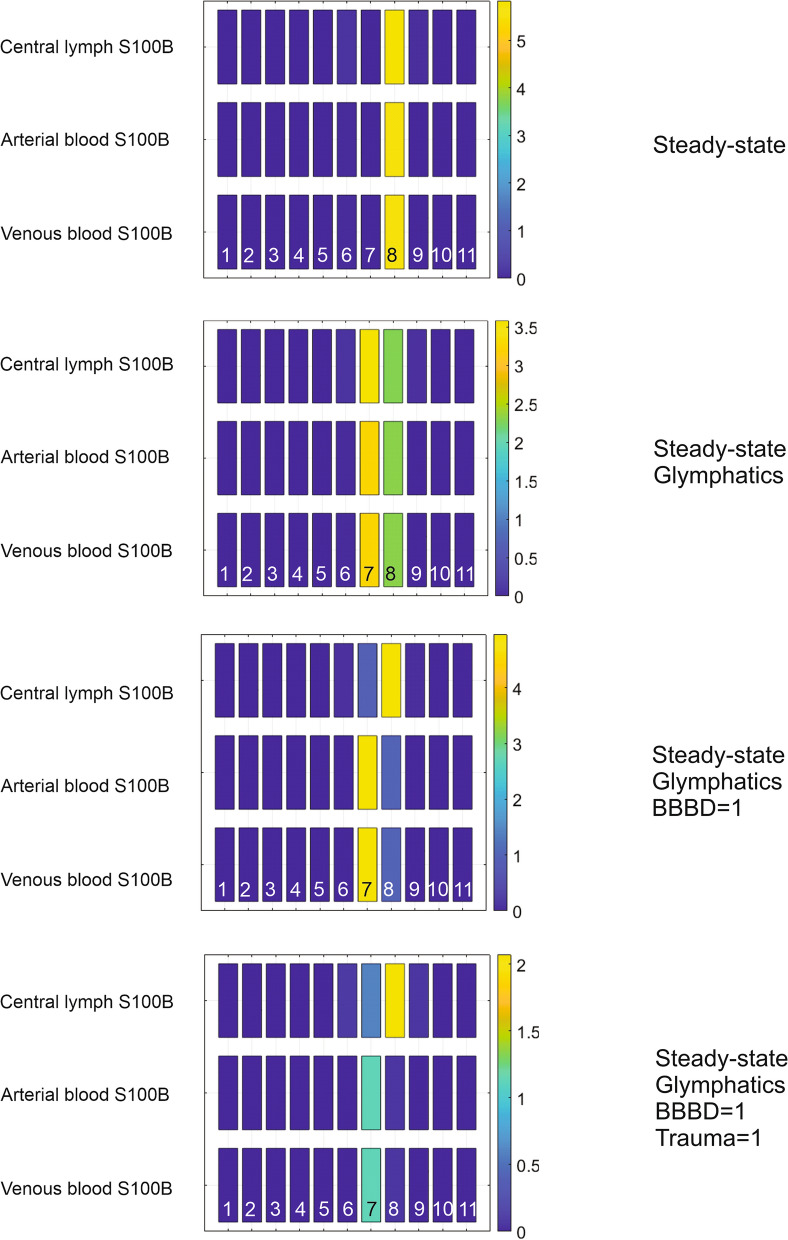
Sensitivity analysis for model 2. Steady-state conditions refer to the sensitivity measured at 10 h of simulation. The x axis in the heatmap plots specifies the sensitivity inputs. Y axes show the normalized sensitivity of venous, arterial, and lymphatic fluid obtained by varying the values of S100B in the compartments indicated by the numbers. The values of the outputs were integrated over time. We used global sensitivity analysis to interpret the impact of S100B levels in various organs on venous, lymphatic, and arterial biomarker levels. The key for the numbers at the *bottom* of each panel is 1: Venous blood; 2: Vascular compartment brain; 3: Vascular compartment adipose; 4: Vascular compartment gut; 5: Vascular compartment muscle; 6: Interstitium adipose; 7: Interstitium brain; 8: Interstitium gut; 9: Interstitium muscle; 10: Arterial blood; 11: Central lymph. When the brain is isolated from the periphery, and the only source of S100B available is the content of peripheral organs (*top panel*), the gut is the chief controller of body fluids S100B. However, when a communication brain to periphery is established via glymphatic drainage, the brain becomes the most influential organ for circulating S100B. This remains true after BBBD and the opening of the communication between the astrocyte content of S100B and the interstitium in the brain. For the brain interstitium S100B, in this simulation we used 10 ng/ml a concentration between CSF values (~ 3 ng/ml) and the measured interstitial value reported in [55]. See also Additional file 1: Fig. S1, Additional file 2: Fig. S2, Additional file 3: Fig. S3

We ran a sensitivity analysis for steady-state values of *Central lymph, Arterial blood S100B*, and *Venous blood S100B*. Under normal conditions (*BBB_Index* = 0), the main contributor to the peripheral fluid signals was gut S100B (Fig. 6). When glymphatics were added to the simulation, the brain contribution to the S100B signal surpassed the gut. When BBB disruption was simulated (*BBB_Index* = 1), the main contributor to the signal in blood remained the brain, but the contribution of gut levels affected *Central lymph* readouts. In addition to BBB disruption, we simulated brain trauma (opening the communication between the glial content of S100B with the brain interstitium): brain interstitial S100B remained the chief contributor to the vascular levels of S100B. The profiles of venous changes under these conditions are shown in Fig. 8A. Note the small but measurable contribution of glymphatic drainage to the venous signal.

**Fig. 7:**
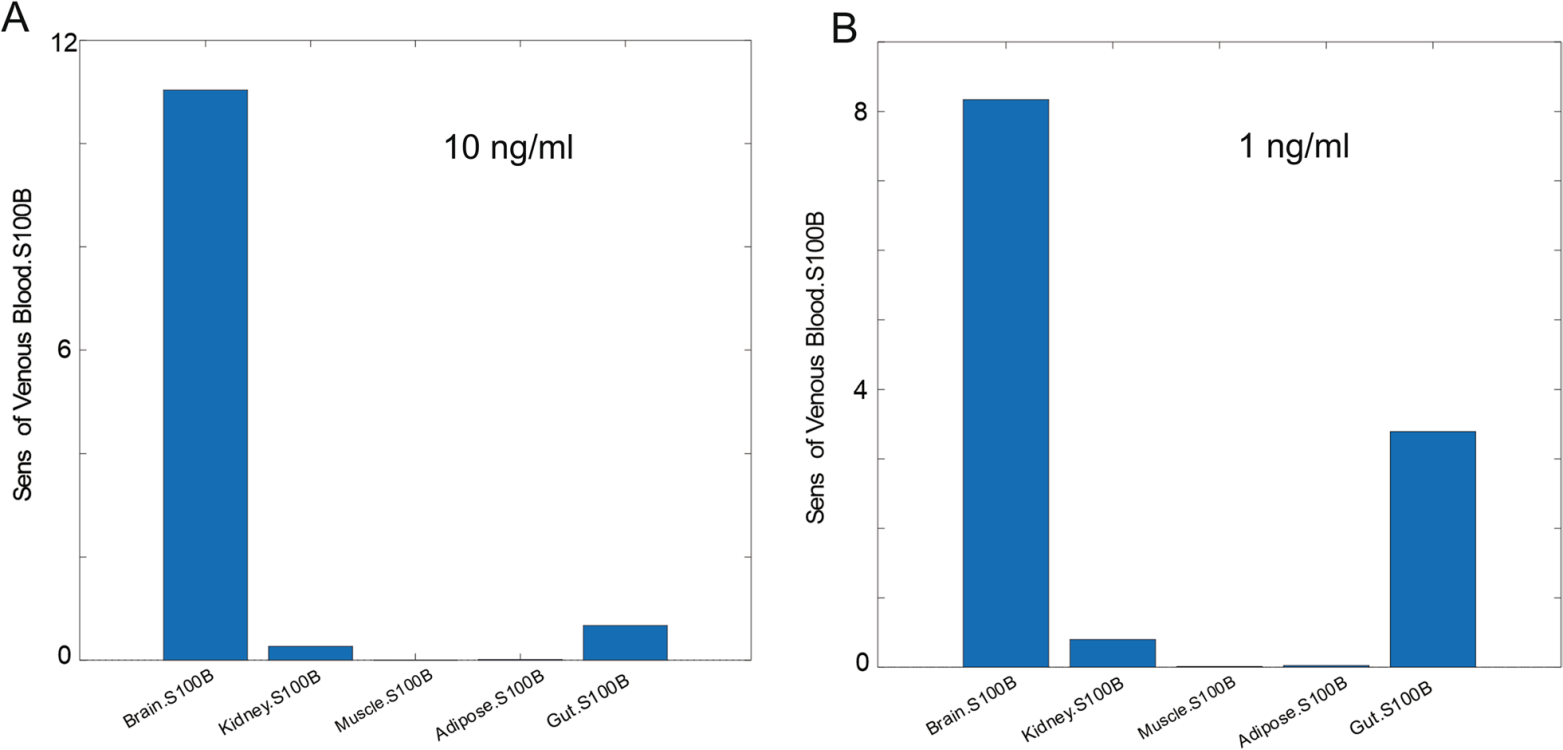
Sensitivity analysis at two initial concentrations of brain interstitial S100B. The only difference obtained by this comparison relates to the increased contribution of gut S100B at lower concentrations. The simulation was run for 25 h. See also Additional file 1: Fig. S1, Additional file 2: Fig. S2, Additional file 3: Fig. S3

**Fig. 8:**
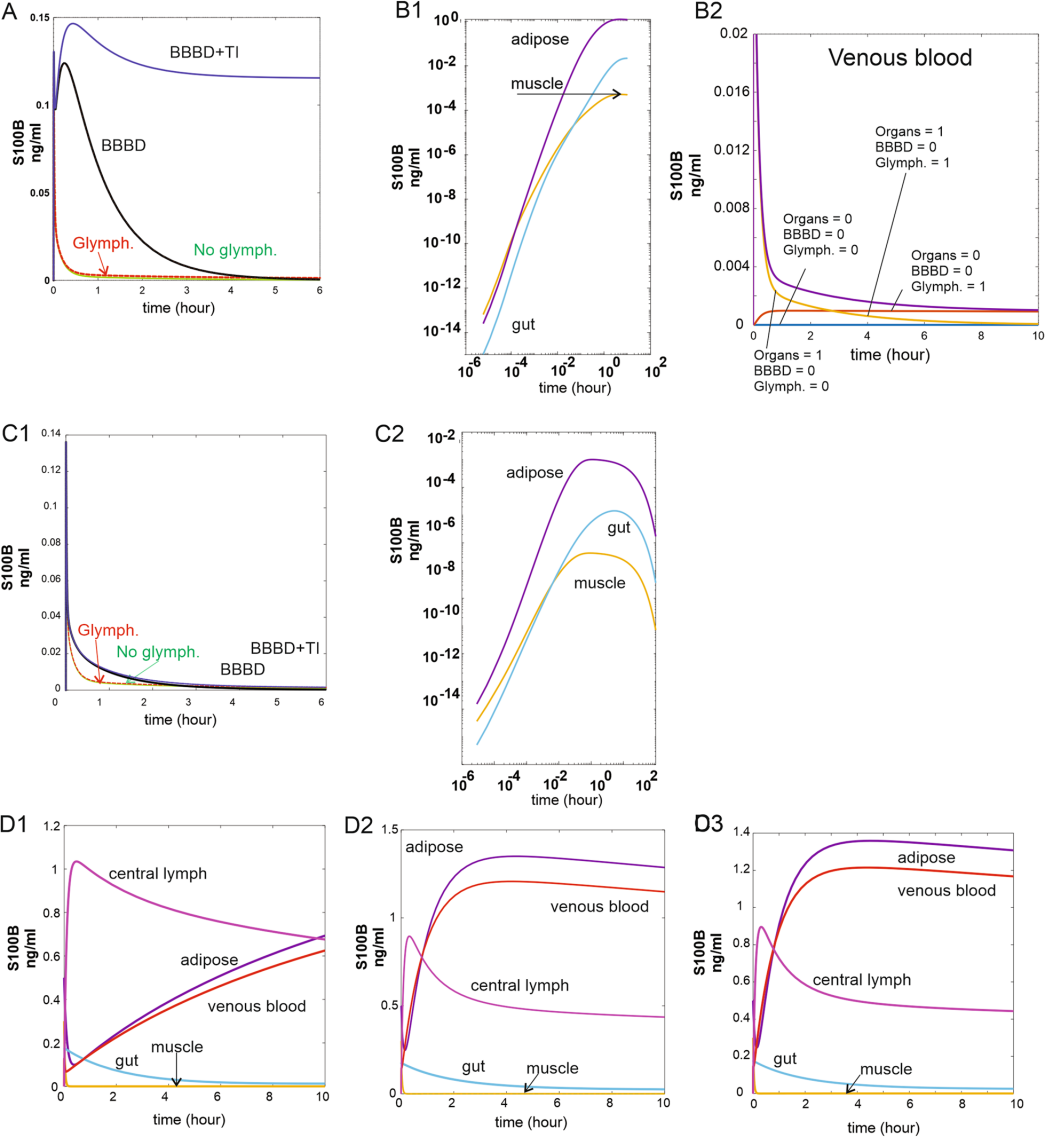
**A** Effects of glymphatics, BBB disruption, and the trauma index on venous blood S100B. Note that activating release from astrocytes (*Trauma_Index*) prevents return of S100B to pre-BBBD values, suggesting that parenchymal S100B is involved in the half-life of S100B. **B1** and **B2** “Filling” of empty organs (S100B set at 0 in adipose, muscle, and gut tissues) after a 100-h simulation with glymphatic communication between brain interstitial S100B and periphery. **B2** Changes in venous S100B in empty or full organs in the presence or absence of glymphatic contribution. When no S100B is available (*blue line*) venous levels are clamped at 0. When S100B in peripheral organs and glymphatic communication are present, a slight transient increase in venous S100B is seen. This is amplified by “opening” the BBB and establishment of a communication between glial cells reservoir and brain interstitial S100B. The data show that peripheral levels in organs can derive from brain reservoirs. **C1** Simulation identical to **A** but at a lower concentration of brain interstitial S100B. **C2** Simulation as in **B1** but with low levels of brain interstitial S100B. **D** Time course of S100B changes in various compartments under normal (**D1**), BBB disruption (**D2**), and trauma (**D3**)

For the sensitivity analysis shown in Fig. 6, we used an interstitial concentration of S100B of 10 ng/ml. This value is of course central to the model since it governs the levels of S100B in peripheral organs and blood under normal conditions or after BBBD or trauma. We rerun the simulation and sensitivity analysis with a low value of interstitial S100B and compared the results with what obtained with 10 ng/ml. The results are shown in Fig. 7; A) refers to 10 and B) to 1 ng/ml S100B in the brain interstitium. Note that no significant differences were seen in overall sensitivity analysis. Similarly, we run a simulation of venous values under various conditions using these two values of interstitial S100B (compare Fig. 8A to C1) to demonstrate an overall reduction of signal in venous blood at low concentrations of S100B, as expected. Additional results for sensitivity analysis are shown in Additional file 1: Fig. S1, Additional file 2: Fig. S2, Additional file 3: Fig. S3.

We previously measured S100B in several peripheral organs (see [3, 4]) and assigned these values as initial conditions for the simulations presented herein (see Tables 1 and 2). We tested the hypothesis that the levels measured in peripheral organs lacking mRNA for S100B were due to diffusion of S100B from the blood. Figure 8B shows these changes with 10 ng/ml interstitial S100B, while Fig. 8C2 refers to 1 ng/ml. We started the initial conditions with all organ levels set arbitrarily at 0 to test the extent of organs’ uptake of circulating S100B. Note (Fig. 8B1) the increase in S100B due to the vascular uptake over a long period of control conditions (*BBB_Index* = 0). Figure 8B2 shows the contribution to the venous levels of glymphatics and when the *BBB_Index* is set to 0. The data in CD1-D3 show the changes in variables when BBBD and trauma were modeled. We then studied the changes in several compartments (Fig. 8D) under the same conditions. Note the effects of BBB disruption (D2) and trauma (D3) to organs and blood. Thus, peripheral organs take up S100B from the circulation to, in turn, contribute to blood levels. The amount of organs’ uptake of S100B depends on the assumed interstitial concentration of S100B.

**Fig. 9:**
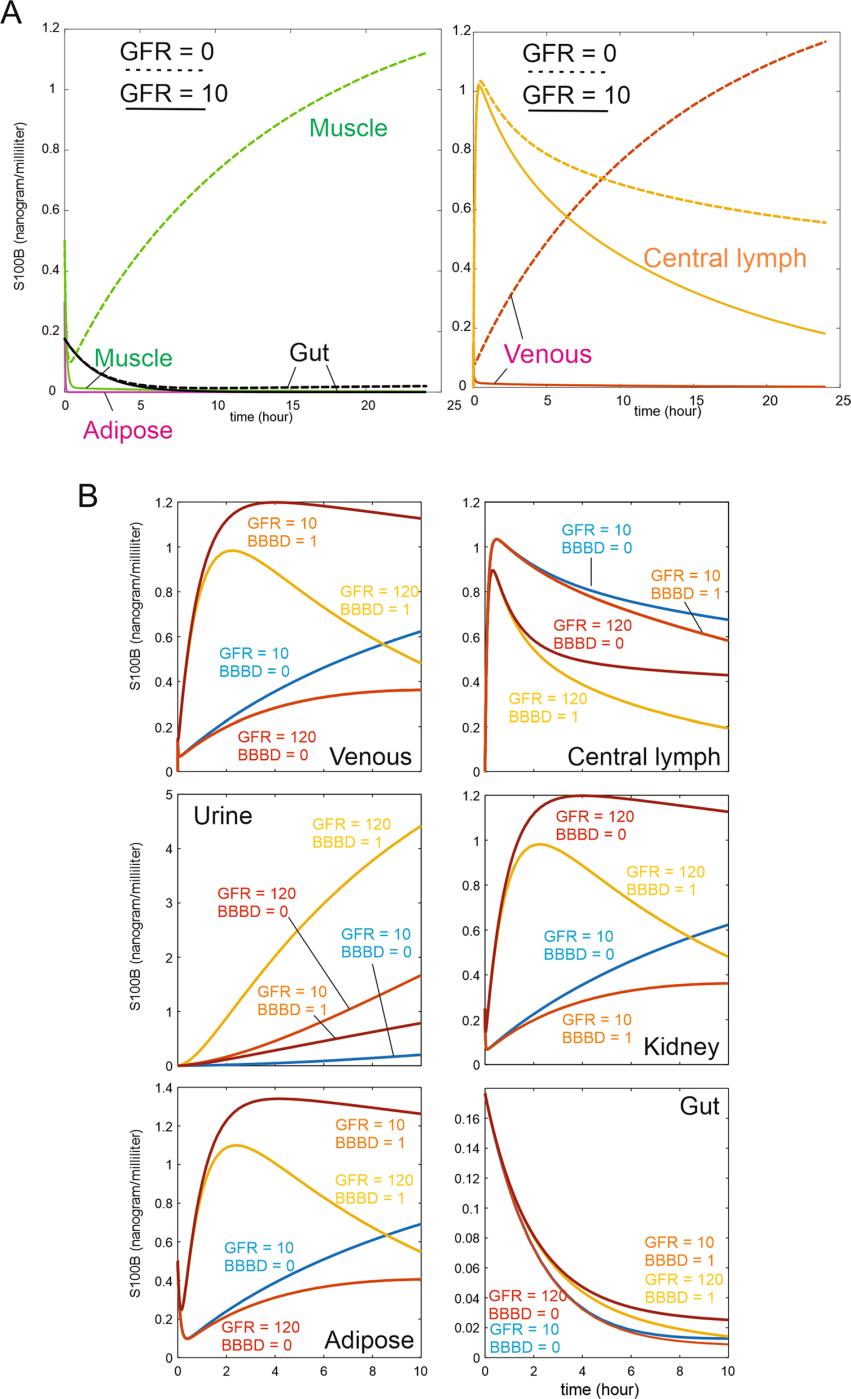
**A** Effects of removing kidney filtration from the model. Note the increase in muscle S100B and venous levels, showing that glomerular filtration rate controls peripheral levels and kinetic behavior of S100B. **B** Effect of varying glomerular filtration on S100B. Note the drop of venous S100B with increased glomerular filtration rate and the lack of effect of GFR on brain interstitial levels and gut S100B. Also note organ-dependent changes in S100B with low or high GFR

The impact of glomerular filtration rate (GFR) and urine formation on blood S100B levels was evaluated (Fig. 9). Under intact or BBBD conditions, GFR greatly influenced the levels of S100B measured in blood, lymphatics, urine, and organs. When setting GFR to zero (Fig. 9A), we found a profound effect of kidney excretion on both organ (*left panel*) and biological fluids (*right panel*). In Fig. 9B, GFR was set at 10 or 100 while also varying *BBB_Index* from 0 to 1.

**Fig. 10:**
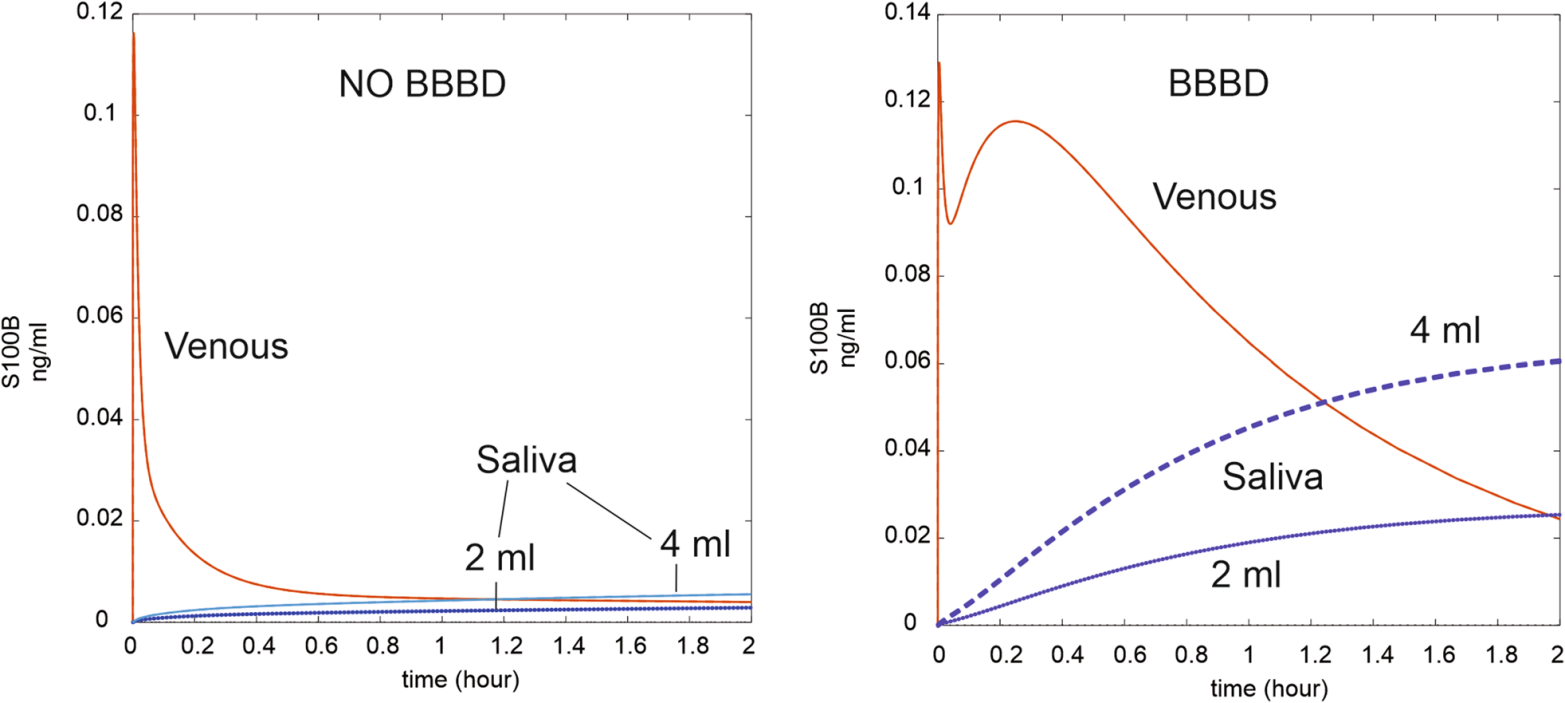
Salivary levels in control or after BBB disruption. Note the delayed progression of salivary S100B towards steady state. Swallowing of saliva was not modeled for clarity and because nothing is known about the half-life of S100B in saliva. See refs. [2, 32, 33]. Saliva production was modeled at 4 and 2 ml/min

Recent reports used salivary S100B and compared its values to venous blood levels [33]. We simulated the passive extravasation of arterial blood to form crevicular fluid [45], see Fig. 10. The levels of S100B in saliva, at steady state, were larger than those in blood when blood flow to saliva was adjusted to 4 ml (upper end of physiologic levels [46]).

**Fig. 11:**
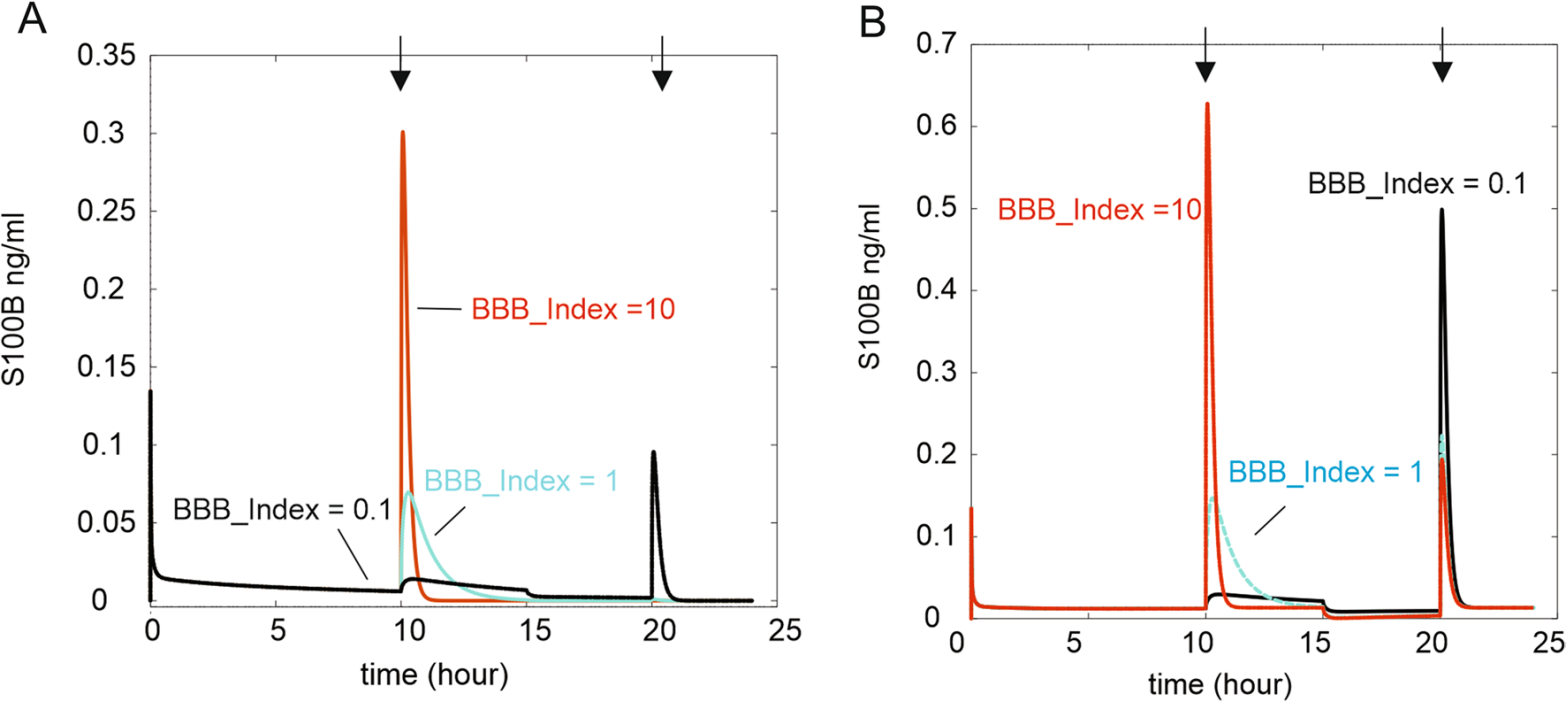
Effect of the previous extent of BBB disruption on secondary BBB insults. Note that a delayed opening of the BBB after an earlier disruption translates into venous S100B levels that are inversely proportional to the extent of the earlier episode. In other words, a supramaximal increase in BBB release of S1000B appears to deplete the brain sources, allowing only a minimal release of S100B by a subsequent episode. See sections “Results” and “Discussion”

We formulated the hypothesis that after BBB disruption the half-life of S100B in blood is determined in part by the availability of S100B in the brain interstitium. This was tested as follows (Fig. 11). We simulated a BBBD after reaching steady state at two time points (*arrows* in Figure). Note that a broad range of *BBB_Index* was explored (indicated in Figure). Also, note that when the *Trauma_Index* was 0, the second BBBD episode had little effect on S100B, unless the first BBBD was minimal (0.1). We then repeated the simulation with *Trauma_Index* set to 1. The secondary BBBD response was restored parallel to a decreased depletion of interstitial S100B in the brain (not shown). This suggests that levels of S100B in the interstitium of the brain are in part responsible for the time-dependent changes in S100B in blood.

## Discussion

The simulation efforts presented herein revealed several surprising findings requiring human trials or animal experiments to be confirmed or refuted. These unexpected results are listed in separate paragraphs below.

### Extracranial sources

It is well known that the distribution of S100B protein is not restricted to the brain. Several extracranial sources have been hypothesized to contribute to the blood levels used clinically [4, 5, 13, 15, 18]. Our simulation revealed that the contribution of “usual suspects” skin, and adipose tissue is relatively minor compared to the impact of gut and muscle release of S100B (Figs. 3 and 6). The possible explanation of these levels of S100B may depend on either local synthesis or uptake from blood. Since mRNA for S100B is lacking in gut tissue [4], the second explanation was tested (Fig. 7B1) by running a simulation where the initial values for organs’ S100B were arbitrarily set to zero while allowing for glymphatic-mediated contribution to blood (see also below). At steady state, peripheral tissues were loaded with levels of S100B comparable to those measured in vivo (Fig. 8A, B1) [3]. While the effects of glymphatics on venous levels was small (Fig. 8A), a prolonged stimulation (100 h) allowed to unveil a powerful effect on organs’ levels of S100B (Fig. 8B1), suggesting that glymphatic connection between brain and periphery was sufficient to load previously depleted organs with the biomarker. Note (B2) that when organs’ levels, BBBD, and glymphatics were set to 0, no venous signal was seen, suggesting that these three parameters are the exclusive contributors to steady-state organs’ levels of S100B. Thus, the most parsimonious explanation for the peripheral presence of S100B is the uptake of circulating protein, as also shown in an animal model [4]. Conversely, these levels remain relatively stable once achieved until an event, such as BBB disruption occurs (Fig. 8). We also run simulations with low levels of interstitial S100B in brain (1 in lieu of 10 ng/ml). No major qualitative differences were seen at lower levels, albeit the responses to BBBD and trauma were reduced.

The question of whether peripheral levels of S100B contribute to the venous signal was answered by simulating control conditions or by adding BBB disruption events (Fig. 8A). At pre-BBBD time points, the brain influenced the blood signal via a mechanism involving glymphatic circulation (see above). BBBD (Fig. 8D2) and the trauma index in D3 dwarfed the control changes in S100B caused by other sources (organs and glymphatics). Sensitivity analysis showed that gut and muscle, but not kidney or adipose tissue, influence venous levels pre-BBBD, but brain contribution dominates after BBB disruption (Fig. 7 and Additional file 1: Fig. S1, Additional file 2: Fig. S2, Additional file 3: Fig. S3). Our results have thus shown that circulating S100B released by glymphatics in lymph and venous fluids is a likely scenario explaining tissue levels in the absence of transcription in peripheral organs. Additional BBB disruption did not increase S100B in organs (Fig. 8D1–D3). We have also shown that serum levels of S100B are only marginally affected by the release of S100B from organs, since the increased venous S100B never approached the cut-off value of 0.1 or 0.15 nanogram/milliliter, which is the clinical ceiling for control subjects [43, 47].

### Do glymphatics contribute to biomarker blood levels?

Please note that as a semantic and scientific explanation of how brain effluxes solutes is still in progress [48–51], the term glymphatics is used here simply as a conveyor of the concept of brain clearance and not as an endorsement of a particular hypothesis. The “glymphatic flow” (in ml/h) may be paravascular or not; the only assumption in the model is that a flux from brain to lymphatic system exists. It was suggested that the primary source of S100B after traumatic brain injury is the brain's communication with blood via glymphatic drainage [36]. We found no significant contribution of glymphatics to the overall signal in blood after BBBD (Figs. 6 and 8D1–D3). However, a small steady-state contribution of glymphatics to the pre-BBBD signal was observed (Fig. 8A). This contribution was however sharply decreased by lowering the interstitial brain S100B levels to 1 ng/ml (Fig. 8C1). At 10 ng/ml interstitial brain values of S100B, this finding suggests a continuous “trickle” of brain protein from the brain extracellular space into the blood via lymphatic drainage under physiological conditions. If this were the case, one expects that levels in blood will continuously increase, which is not what has been shown in human subjects. A fraction of what is being released from the brain is taken up by peripheral tissues as discussed above, but the factor that fully counteracts this constant source of S100B is kidney excretion of S100B (Fig. 10). In fact, when GFR was set to zero (in A), a constant increase was observed in peripheral fluids and organs. Thus, an equilibrium exists between glomerular filtration of small molecular weight protein [3] and S100B release from the brain interstitium via the glymphatic system. This finding predicts that patients with reduced glomerular filtration rate may have elevated levels of S100B (and other biomarkers) in the absence of a BBB contribution. A recent study [31] has shown that a constant source of S100B from brain to blood exists. This is an indirect validation of our modeling effort.

### Effects of parenchymal trauma on biomarker’s levels

Brain damage and BBB disruption contribute to the overall levels of S100B in blood [34, 35]. However, in clinical practice is impossible to dissect out the contributions of these two factors independently. We have developed a subroutine in our software model that allows us to quantify and describe these two sources of blood S100B (Fig. 11). We ran a simulation where levels of blood S100B in response to two BBB disruption events were monitored. When the *Trauma_index* was set to zero (no contribution of cellular release of S100B on peripheral or brain interstitial levels), we noted that the second BBB disruption episode did not cause an increase of S100B in venous blood unless a minimal level of disruption (*BBB_Index* = 0.1) was used for the first event. We monitored the reserve of S100B sources in the interstitium to show that depletion of interstitial S100B occurred after the first, more significant, episodes. Therefore, the subsequent BBB “opening” was consequential only if a minimal depletion of S100B occurred during the first episode. When the *Trauma_Index* was set to 1, replenishment of S100B in the extracellular space of the brain was repristinated, allowing for S100B release after the second BBB disruption event. This is a potentially important finding since it suggests that astrocytic sources of S100B are crucial in controlling the extent and duration of S100B during BBB disruptions.

### Comparison with existing models of blood biomarkers

In addition to our own prototype model [3, 32, 52], an effort to mimic biomarkers’ fate after TBI has been published [53]. The Authors use a much simplified, one-compartment model derived from oral absorption of therapeutic drugs. The limitations we found to be most relevant compared to the present study are: (1) Lack of distribution variables. Because only one compartment is used, the marker undergoes only blood distribution and thus disallows understanding of the impact of extracranial sources or the uptake of the marker by organs; (2) The model has only one path for the biomarker to leave the brain, ignoring glymphatic drainage; (3) Being a single-compartment model, there is no effort to reproduce organ size (including the brain) or cerebral and organ blood flow; and (4) The excretion data are presented only as a means to balance brain release.

### Brain levels of S100B

We initially used a middle-of-the-road concentration of S100B in brain (10 ng/ml). This value is supported by a recent paper where interstitial S100B levels were measured in brain slices [54]. Much higher levels have been measured after stroke and TBI [55, 56]. The pathological levels of S100B in brain tissue were modeled by the *Trauma_Index,* which provides a replenishment of brain S100B by release from a reservoir with 50 ng/ml S100B. We already presented and discussed the outcome of trauma on S100B levels after BBBD. We, however, also explored the possibility that under normal conditions, S100B levels in brain interstitium are equal to those typically reported for cerebrospinal fluid [57, 58]. When results with 10 vs 1 ng/ml were analyzed, no qualitative differences were found in terms of sensitivity or overall S100B dynamics (Fig. 7, Additional file 1: Fig. S1, Additional file 2: Fig. S2, Additional file 3: Fig. S3). However, the responses to BBBD and trauma were greatly reduced (compare 8A to 8C1). Thus, the results from our simulation were independent from the levels of S100B in brain used as initial conditions.

### Limitations

The main limitation of our study is that we did not attempt to adapt the model to existing data on S100B, except when using realistic quantities of S100B in peripheral organs and a comparison of data with a previously established control value ceiling. All the data sets available to us report S100B values in venous blood in individuals affected by a certain pathology or control subjects. In a previous work [3, 32] we focused on these pathophysiologic conditions. In the current study, the independent variable is time. To our knowledge, only a few studies reported the time course of S100B in blood; usually, only 2–3 time points were published. This makes it impossible to directly validate our model with existing data. We also used blood flow and volumetric data from the literature and accepted equilibrium values derived from the simulation of protein distribution after local injection [37]. Therefore, our modeling effort was not geared toward reproducing existing data but rather to allow for a discovery process of mechanisms that human subject-derived data make impossible to study.

### Future directions

The open-source format of the software developed herein (available at https://www.mathworks.com/matlabcentral/fileexchange/106145-diagnostic-pbpk-model-for-s100b?s_tid=srchtitle_Damir_2) will enable other researchers to adapt the core model to other situations and answer questions perhaps related to other biomarkers. Future developments will add the effect of molecular size (molecular weight and radius; see) on the movement across different compartments. This was already done in an older version of this model [3]. The main changes due to molecular size are likely to affect kidney filtration, passage across the BBB, and the overall kinetic properties of the marker. As new markers of CNS function are unveiled, we will focus on the physical and chemical properties of these proteins related to the voyage across organs and biofluids. In addition, we will develop a model that considers other biological variables, such as sex and age [3]. Finally, the model based on human subjects can be allometrically manipulated to include laboratory animals which are often used as surrogate experimental targets.

## Supplementary Information

**Additional file 1: Fig. S1.** Comparison of sensitivity analysis at two levels of brain interstitial S100B. The plot refers to time-dependent sensitivity. Note that unexplained variance was 0 in both simulations, demonstrating that the changes shown explain the variance of the model simulation. First and total order Sobol indices for model responses are shown.

**Additional file 2: Fig. S2.** Comparison of sensitivity analysis at two levels of brain interstitial S100B. The plot refers to time-dependent sensitivity. Note that unexplained variance was 0 in both simulations, demonstrating that the changes shown explain the variance of the model simulation. First and total order Sobol indices for model responses are shown.

**Additional file 3:** **Fig. S3.** Bar graph of the results shown in 1 and 2. See [40].

## Data Availability

The model is currently available online.
